# MOBCA: Multi-Objective Besiege and Conquer Algorithm

**DOI:** 10.3390/biomimetics9060316

**Published:** 2024-05-24

**Authors:** Jianhua Jiang, Jiaqi Wu, Jinmeng Luo, Xi Yang, Zulu Huang

**Affiliations:** 1 Center for Artificial Intelligence, Jilin University of Finance and Economics, Changchun 130117, China; cmwm2000@gmail.com (J.W.); 5221191007@s.jlufe.edu.cn (J.L.); acey_yang@163.com (X.Y.); 2 Jilin Province Key Laboratory of Fintech, Jilin University of Finance and Economics, Changchun 130117, China; 3 College of Foreign Languages, Jilin Agricultural University, Changchun 130118, China; huangzulu2024@163.com

**Keywords:** evolutionary algorithm, multi-objective optimization, heuristic algorithm, meta-heuristic

## Abstract

The besiege and conquer algorithm has shown excellent performance in single-objective optimization problems. However, there is no literature on the research of the BCA algorithm on multi-objective optimization problems. Therefore, this paper proposes a new multi-objective besiege and conquer algorithm to solve multi-objective optimization problems. The grid mechanism, archiving mechanism, and leader selection mechanism are integrated into the BCA to estimate the Pareto optimal solution and approach the Pareto optimal frontier. The proposed algorithm is tested with MOPSO, MOEA/D, and NSGAIII on the benchmark function IMOP and ZDT. The experiment results show that the proposed algorithm can obtain competitive results in terms of the accuracy of the Pareto optimal solution.

## 1. Introduction

With the advent of the era of artificial intelligence, the evolutionary algorithm of swarm intelligence has received more and more attention [1]. Swarm intelligence algorithms are designed by researchers who are inspired by natural phenomena or population activities [2]. These algorithms are often used to solve complex optimization problems [3].

Optimization problems can be divided into single-objective optimization problems and multi-objective optimization problems [4]. Single-objective optimization problems have more specific objectives than multi-objective optimization problems [5]. Therefore, the solution to multi-objective optimization problems is more complicated. The result of solving a multi-objective optimization problem requires a decision maker to choose the final result [6]. Therefore, when solving multi-objective optimization problems, multiple different results are required for decision-makers to choose. There are usually two ways to deal with multi-objective problems: priori and posteriori [7,8].

A priori means that the decision-maker first weights the decision variables of the problem to be optimized. In this way, the multi-objective problem can be transformed into a single-objective problem [9]. In this case, the problem can be solved by employing a single-objective optimization algorithm. As a result, only one solution can be obtained as the optimal solution. The optimizer must be run multiple times if multiple optimal solutions are required [10]. On the contrary, when dealing with multi-objective problems, the posterior method ensures that the nature of the problem does not change. In other words, the problem will not be transformed into a single-objective problem [11]. This enables the design parameters or conditions to vary within a certain range while obtaining a different set of solutions. Then, the decision maker chooses a solution from the obtained solution set as the optimal solution according to his own preference or actual situation [12].

The multi-objective optimizer aims to find a set of non-dominated solutions, obtain more non-dominated solutions through iteration, and improve the quality of the solution [13]. The resulting non-dominated solution can trade-off between multiple objectives [14]. Compared with adopting a priori method to solve it, the posterior method retains more original characteristics of the multi-objective problem, meaning the method can obtain more optimal solutions in a short time [15].

There are many multi-objective optimization algorithms in the literature that solve multi-objective problems. Most of the algorithms are inspired by the single-objective algorithm, such as the grey wolf optimizer (GWO) [16], particle swarm optimization (PSO) [17], the ant lion optimization algorithm (ALO) [18], the grasshopper optimization algorithm (GOA) [5], the genetic algorithm (GA), and the evolutionary algorithm (EA). These algorithms are summarized in the following Table 1:

Many meta-heuristic multi-objective optimization algorithms (MOAs) have been proposed according to the literature review above. There are many different types of MOAs to solve different types of multi-objective optimization problems (MOPs). The existing MOAs are faced with low speed of convergence [31], are easily trapped by local optima [32,33], lack diversity in solutions when solving MOPs [34,35], etc. Based on the single-objective besiege and conquer algorithm (BCA), we propose an MOBCA to solve these problems. The contributions can be summarized as follows:

(i) The single-objective BCA outperforms other algorithms in the literature, and converging to the local optima is not easy. Therefore, we attempt to demonstrate its effectiveness in MOPs.

(ii) The BCA has been equipped with a grid mechanism to guarantee the diversity in the convergence process.

The rest of the paper is organized as follows: Section 2 provides a literature review concerning MOPs. Section 3 proposes the multi-objective besiege and conquer algorithm. Section 4 presents, discusses, and analyzes the experiment results on the ZDT, IMOP, and real-world problems. Finally, Section 5 concludes the work and suggests future works.

## 2. Literature Review

### 2.1. Multi-Objective Optimization Problem

Multi-objective optimization is a classic optimization problem. This problem needs to optimize multiple objectives at the same time. Therefore, the algorithm is required to find the maximum or minimum of multiple objective functions at the same time [9]. Compared with single-objective optimization problems, only one objective function needs to be optimized. As a result, we can easily compare the pros and cons of the fitness value in single-objective optimization problems.

A multi-objective optimization problem can be defined as follows:

$$\begin{array}{cc} \;\mathrm{Minimize}\;: & F\left(\vec{x}\right)=\left\{f_{1}\left(\vec{x}\right),f_{2}\left(\vec{x}\right),\ldots ,f_{o}\left(\vec{x}\right)\right\}\\ \;\mathrm{Subject}\;\mathrm{to}\;: & g_{i}\left(\vec{x}\right)\geq 0,\;i=1,2,\ldots ,m\\ & h_{i}\left(\vec{x}\right)=0,\;i=1,2,\ldots ,p\\ & L_{i}\leq x_{i}\leq U_{i},\;i=1,2,\ldots ,n \end{array}$$

where *n* is the number of variables, *o* is the number of objective functions, *m* is the number of inequality constraints, *p* is the number of equality constraints, $g_{i}$ is the $i_{th}$ inequality constraint, $h_{i}$ indicates the $i_{th}$ equality constraint, and $\left[L_{i},U_{i}\right]$ are the boundaries of the $i_{th}$ variable.

**Figure 1:**
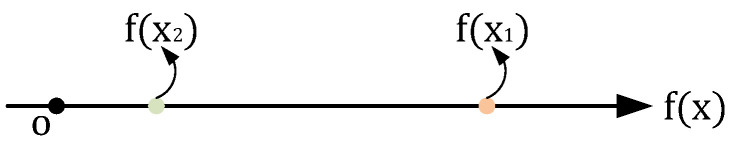
Single-objective optimization problem.

In Figure 1, we obtain two different solutions, $x_{1}$ and $x_{2}$, which correspond to different evaluation values, $f(x_{1})$ and $f(x_{2})$. We can clearly label these two fitness values on a one-dimensional coordinate axis. In this coordinate axis, point *o* is the origin. We can clearly understand the relationship between the fitness values corresponding to the two solutions. That means, if we need to determine the smallest fitness value for the problem, solution $x_{2}$ in the figure is desirable.

**Figure 2:**
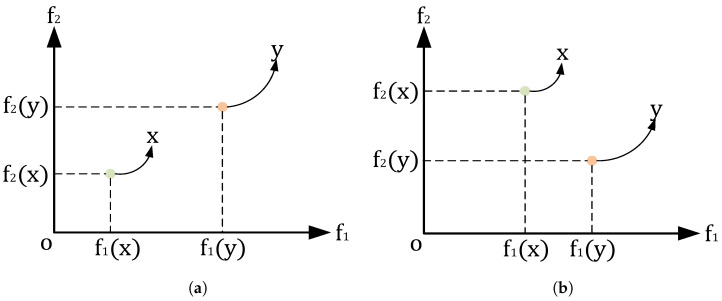
Different situations in a multi-objective problem (for example: two objectives). (**a**) Situation 1: solution *x* dominates *y*. (**b**) Situation 2: *x* and *y* do not dominate each other.

For fitness values in multi-objective problems, each evaluation process produces multiple evaluation values. This means that each evaluation process will produce at least two evaluation values. This is shown in Figure 2a,b. For example, during the two evaluations processes, two sets of fitness values are generated as follows:

$$\begin{array}{c} Fitness\_Set_{1}=\left\{f_{1}\left(x\right),f_{2}\left(x\right)\right\},\\ Fitness\_Set_{2}=\left\{f_{1}\left(y\right),f_{2}\left(y\right)\right\}, \end{array}$$

where *x* is the first solution of Fitness_set, $f_{1}$ is the first evaluation function, $f_{1}\left(x\right)$ is the evaluation function value obtained by *x* through the first evaluation function, and $f_{2}$ and $f_{2}\left(x\right)$ are the same. *y* is the second solution of Fitness_set, its evaluation functions $f_{1}$ and $f_{2}$ are the same as in solution *x*, but the obtained evaluation value is different.

In Figure 2a, all the fitness values of the solution *x* are smaller than the fitness values of solution *y*. From this, we can easily judge that *x* is a better solution than *y*.

In Figure 2b, we can observe that $f_{1}\left(x\right)<f_{1}\left(y\right)$ and $f_{2}\left(x\right)>f_{2}\left(y\right)$, and it is difficult for us to judge whether *x* or *y* is better.

As mentioned above, the evaluation of the solution is more complicated than that of the single-objective problem. When we evaluate a multi-objective solution, it is difficult to determine an evaluation standard to judge the quality of the solution. So, the introduction of a series of definitions of Pareto is necessary [36].

**Definition** **1.** *Pareto Optimality:*

*Solution $\vec{x}\in X$ is deemed Pareto-optimal if and only if the following is applicable:*

$$\{∄\vec{y}\in X|F\left(\vec{y}\right)≺F\left(\vec{x}\right)\}.$$

**Definition** **2.** *Pareto Dominance:*

*It is assumed that there are two vectors: $\vec{x}=\left(x_{1},x_{2},...,x_{k}\right)$ and $\vec{y}=\left(y_{1},y_{2},...,y_{k}\right)$. Vector $\vec{x}$ dominates vector $\vec{y}$ (denoted as x≻y) if and only if the following is applicable:*

$$\forall i\in \left\{1,2,\ldots ,k\right\}:f_{i}\left(\vec{x}\right)\leq f_{i}\left(\vec{y}\right)\wedge \exists i\in \left\{1,2,\ldots ,k\right\}:f_{i}\left(\vec{x}\right)<f_{i}\left(\vec{y}\right).$$

**Definition** **3.** *Pareto-optimal Set:*

*The set including all Pareto-optimal solutions is called a Pareto set:*

$$P_{s}:=\left\{\vec{x},\vec{y}\in X|∄F\left(\vec{y}\right)≺F\left(\vec{x}\right)\right\}.$$

**Definition** **4.** *Pareto-optimal Front:*

*A set that contains the value of objective functions for the Pareto solutions set:*

$$P_{f}:=\left\{F\left(\vec{x}\right)|\vec{x}\in P_{s}\right\}.$$

**Figure 3:**
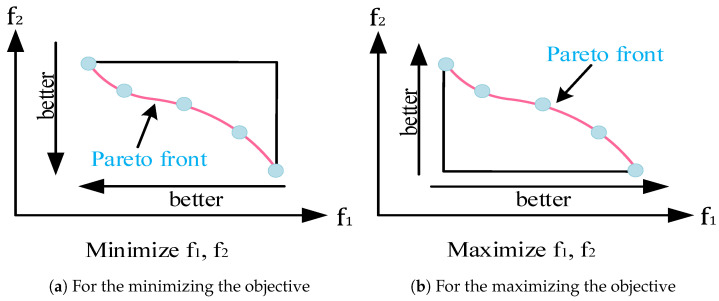
Pareto fronts for different optimization directions.

In Figure 3a,b, all solutions that lie on the Pareto front can dominate solutions that do not. The set of points located on the Pareto front constitutes a Pareto optimal solution set. This solution set is the optimal solution to the multi-objective optimization problem.

### 2.2. Multi-Objective Optimization in Metaheuristics

The ultimate goal of a multi-objective optimization algorithm is to determine a Pareto optimal solution set and ensure that the solutions within this set are uniformly distributed across the Pareto front [37]. A solution set like this can provide decision-makers with more high-quality choices. Therefore, when solving multi-objective optimization problems, we mainly focus on two issues: the diversity of the solution set and the distance between the solution and the true Pareto front [38].

Metaheuristic optimization algorithms can perform well in solving multi-objective optimization problems [39]. The meta-heuristic algorithm usually uses a random strategy to generate populations and adaptively adjusts the position of randomly generated offspring, which can converge to cover the Pareto front. The parallelism and scalability of the heuristic algorithm are also noteworthy, enabling it to solve large-scale and complex multi-objective optimization problems.

In recent decades, multi-objective optimization algorithms have received extensive attention in their field due to their powerful ability to solve complex optimization problems [40]. Some novel and excellent multi-objective optimization algorithms have been proposed: the multi-objective grey wolf optimizer (MOGWO) [8], the multi-objective ant lion optimizer (MOALO) [11], the multi-objective whale optimization algorithm (MOWOA) [20], the vector-evaluated genetic algorithm (VEGA) [30], the niched Pareto genetic algorithm (NPGA) [28], the non-dominated sorting genetic algorithm (NSGA) [22], the non-dominated sorting genetic algorithm II (NSGA-II) [23], the non-dominated sorting genetic algorithm III (NSGA-III) [24], and the multi-objective evolutionary algorithm based on decomposition MOEA/D [26]. The above algorithms can approach the real Pareto optimal frontier in some multi-objective problems. However, according to the NFL theorem, there may be problems that these algorithms cannot solve [41]. So, new multi-objective algorithms should be proposed. In the next section, a new multi-objective optimization algorithm is proposed to solve the multi-objective optimization problem.

## 3. Multi-Objective Besiege and Conquer Algorithm (MOBCA)

### 3.1. Besiege and Conquer Algorithm (BCA)

The BCA algorithm was proposed by Jiang et al. in 2023. To enable BCA to solve multi-objective problems, the single-objective version of BCA also deserves a brief introduction here. The process of BCA optimization and the location update process can be briefly summarized by the following mathematical formulas:

(1) $$S_{j,d}^{t+1}=\left\{\begin{array}{cc} B_{d}^{t}+\left|A_{r,d}^{t}-A_{i,d}^{t}\right|\times k_{1} & \;,\;if\;rand<BCB,\\ A_{r,d}^{t}+\left|A_{r,d}^{t}-A_{i,d}^{t}\right|\times k_{2} & ,\;else, \end{array}\right.$$

where $S_{j,d}^{t+1}$ is the $j_{th}$ soldier of the $d_{th}$ dimension with ${\left(t+1\right)}_{th}$ iteration, $B_{d}^{t}$ is the current best army with $t_{th}$ iteration, $A_{i,d}^{t}$ is the $i_{th}$ army of the $d_{th}$ dimension with $t_{th}$ iteration, $A_{r,d}^{t}$ is a random army of the $d_{th}$ dimension with $t_{th}$ iteration, and $k_{1},k_{2}$ is the disturbance coefficient.

$k_{1}$ and $k_{2}$ can be calculated using following two equations:

(2) $$k_{1}=sin\left(2\times \pi \times rand\right),$$

(3) $$k_{2}=cos\left(2\times \pi \times rand\right).$$

Parameter BCB is set to adjust global search or local search. It can be dynamically changed according to the distance between the current army and the best army. If the current army is the best army in the population, then parameter BCB will change to 0.2 in order to enhance the global search. This will avoid local stagnation.

The BCA algorithm starts the optimization process with a set of randomly generated populations. In the optimization process, the initial population is 1/3 of the set population size, which means that each army has three soldiers. During the position update process, each army will generate three soldiers according to the position update Equation (1). But, the positions of these soldiers will only be stored when the army is updated. The soldiers’ position will be eliminated if the fitness value of soldiers is not better than that of armies when the evaluation process is completed. When all armies are updated, the algorithm judges the distance relationship between the current army and the global optimum. So, the algorithm can adjust the value of BCB according to the distance, which can control the global search or local search. This is an aggressive position update strategy, and it also enables BCA to have better global optimization capabilities.

The number of function evaluations (FEs) is described as Equation (4).

(4) FEs=FEs+nSoldiers×nArmies,

where nSoldiers is the number of soldiers in each army, and nArmies is the number of armies. The algorithm will output the best solutions when the FEs reaches max function evaluation times (Max_FEs).

### 3.2. Multi-Objective Besiege and Conquer Algorithm (MOBCA)

To apply BCA to solve multi-objective optimization problems, we need to introduce related mechanisms. These mechanisms function roughly as MOPSO [19], including grid, archive, and leader selection mechanisms.

The purpose of the grid mechanism is to determine the location of each solution in the objective space. During the initialization phase, the objective space is divided into several grids according to the scalar parameter div. As the optimization process proceeds, more solutions are obtained by the MOAs. Suppose that a solution is not located in the grid at one iteration, the grid will be updated to cover the new solution. Specifically, the grid is used to determine the relative positions between solutions in objective space. This information will be used to determine whether a solution is retained. This is associated with the archive. In the literature, MOGWO utilizes the grid mechanism to decide the α,β, and δ wolf [8]. A solution located in the sparest grid will be chosen as the α wolf, while the solutions in the second- and third-sparest grids will be chosen as the β and δ. This method aims to lead the algorithm to maintain diversity in solutions.

The archive stores non-dominated solutions during iterations. The maximum size of the archive is equal to the population size. In each iteration, the archive members will be compared with the new offspring population. Then, the archive will update the latest non-dominated solutions as the new archive members. In the replacement process, the archive size may exceed the maximum limitation. The grid mechanism comes in handy to maintain the diversity of the non-dominated population. The grid location will be rearranged if the archive size exceeds the maximum limitation. The surplus member will be deleted via the information on the crowding degree. Those solutions that have occupied a crowding grid will be more likely to be deleted. In GWO, the algorithm only stores the first three wolves in the population [16]. However, this is not suitable in the MOA. We expect to obtain more diverse solutions to guide the subsequent optimization process. So, an archive equal to the population size to store the solutions is necessary.

In PSO, the iterative update of particles is based on global best and personal best [17]. In MOPs, the personal best is easily updated if the new solution dominates the old one. In contrast, the global best is hard to update. It is easy to select a leader in single-objective optimization to guide the population to a promising area to approach the global optimum. However, in multi-objective optimization, comparing one solution with another makes it hard due to the Pareto optimal concepts mentioned before. Therefore, we must use a leader selection mechanism to solve this problem. In multi-objective problems, promising solutions approach the Pareto front and have good population diversity. The solution in the archive set is composed of many promising solutions. So, the leader selection mechanism will use the roulette method to select the least crowded solution in the archive as the leader.

The BCA is introduced in the archive mechanism to store non-dominated solutions. The global best solution will be selected from the archive to lead the population. In MOBCA, every soldier may be assigned to a different global best. This will guarantee diversity in the population. Each soldier will move in a different direction to the Pareto Front. Once armies exceed the maximum size, redundant armies will be deleted on the basis of the grid mechanism. The convergence of the MOBCA is guaranteed because it employs the same mathematical model of BCA. During the optimization process, BCA completes convergence by changing the position of the agent factor. This behavior guarantees the convergence ability of an algorithm in the search process according to [42]. The MOBCA inherits all the characteristics of the BCA, which means that the search agents explore and exploit the search space in the same manner.

The main difference is that the MOBCA is based on a set of archive members, while the BCA only saves and improves three of the best solutions.

**Figure 4:**
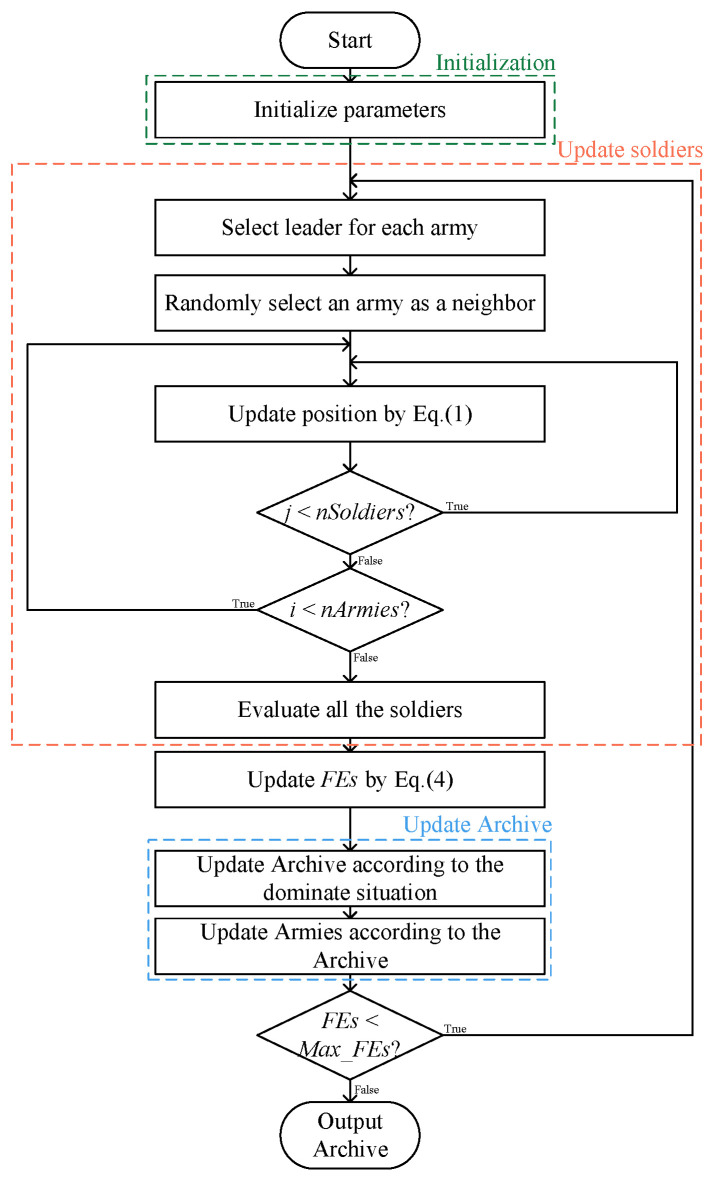
The workflow of the BCA algrotihm.

The workflow of the BCA is shown in Figure 4, and its pseudo code is presented in Algorithm 1.
**Algorithm 1:** MOBCA: Mutil-objective besiege and conquer algorithm.
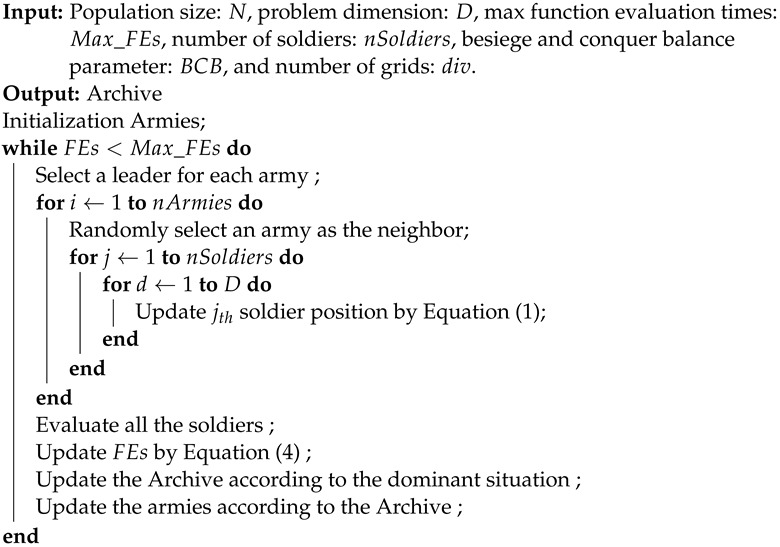


### 3.3. Computational Complexity

The complexity of the proposed MOBCA is based on the number of decision variables, the objective variables, and the population size. Imagine a multi-objective problem with *D* decision variables, *M* objective variables, and *N* particles as an example. The MOBCA mainly includes updated soldiers, an updated archive, and updated armies.

The complexity of update soldiers is decided by N,D, and Max_FEs. This process will be executed Max_FEs/N times; thus, the computational complexity of this process is O(D×N×Max_FEs/N).

The updated archive involves deleting redundant solutions, and the complexity is $O(M\times N^{2}\times Max\_FEs/N)$.

In the MOBCA, after the update archive process, we must also update armies for the next generation, so this process is similar to the update archive. The complexity of this process is $O(M\times N^{2}\times Max\_FEs/N+nArmies\times Max\_FEs/N)$.

Since the D,M, and nArmies are far lower than *N*, the final complexity of the proposed MOBCA is $O(Max\_FEs/N\times N^{2})$.

## 4. Experimental Settings

The experiment codes are executed in a MATLAB R2022b environment under the Windows 10 operating system. All simulations are carried out on a computer with an Intel(R) Core(TM) i7-8750H CPU @ 2.20 GHz 2.21 GHz and memory of 16 G.

### 4.1. Experimental Settings

The proposed algorithm is compared with three well-known algorithms, including the multi-objective particle swarm optimizer (MOPSO) [19], the non-dominated sorting genetic algorithm III (NSGAIII) [24], and the multi-objective evolutionary algorithm based on decomposition (MOEA/D) [26]. The default parameters in PlatEMO v4.3 [43] are used.

Different from the evaluation of a single-objective optimization algorithm, the performance evaluation of a multi-objective optimization algorithm needs to be evaluated by other calculation methods. The specific calculation method of the performance evaluation index is as follows:

For the inverted generational distance (IGD) for measuring convergence [44], its mathematical formula can be expressed as Equation (5):

(5) $$\begin{array}{c} \;\mathrm{IGD}\;=\frac{\sqrt{\sum_{i=1}^{nt}{\left(d_{i}\right)}^{2}}}{n}, \end{array}$$

where nt shows the size of the true Pareto optimal solutions set, and $d_{i}$ indicates the Euclidean distance (ED) between the true Pareto optimal solutions set and obtained solution set. *n* represents the number of obtained Pareto solutions.

IGD can evaluate the distance between the Pareto optimal solution and the actual obtained solution. However, evaluating the sparsity of the Pareto solution set requires the use of another evaluation indicator: hypervolume (HV).

Hypervolume is a widely employed performance metric in the domain of multi-objective optimization [45,46,47]. It quantifies the hypervolume enclosed by a set of solutions within the objective space, representing the volume of the space dominated by these solutions. HV serves as a crucial indicator for assessing the quality of solution sets generated by different multi-objective optimization algorithms, where larger HV values typically indicate superior performance.

The calculation of HV involves the determination of the volume within the Pareto front formed by the set of solutions. It is computed as the integral of the dominated portion of the objective space. Formally, the hypervolume (HV) is defined as follows:

Hypervolume (HV) is calculated using the following Equation (6):

(6) $$HV\left(X\right)=\int_{R^{m}}H\left(X,z\right)\;dz,$$

where *X* represents the set of solutions, $R^{m}$ is the objective space, and H(X,z) is the hypervolume contribution Equation (7):

(7) $$H\left(X,z\right)=\max_{x\in X}\prod_{i=1}^{m}\max\left(0,z_{i}-x_{i}\right).$$

Here, z is a reference point in the objective space. The integral spans the entire objective space, and the computation involves evaluating the contribution of each solution in *X* to the overall hypervolume.

**Figure 5:**
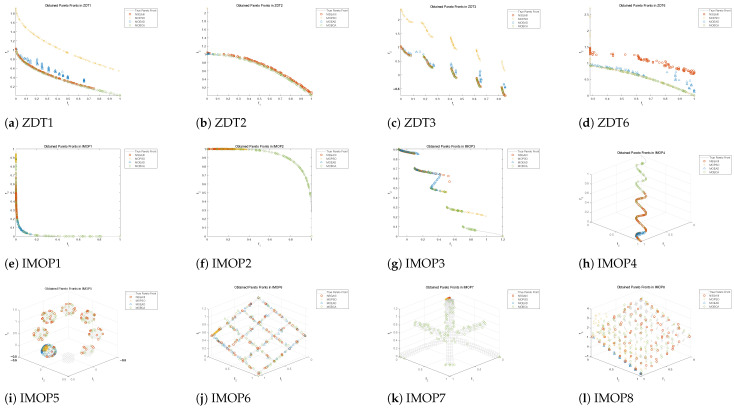
Obtained Pareto fronts by MOBCA, MOPSO, MOEA/D, and NSGAIII.

**Figure 6:**
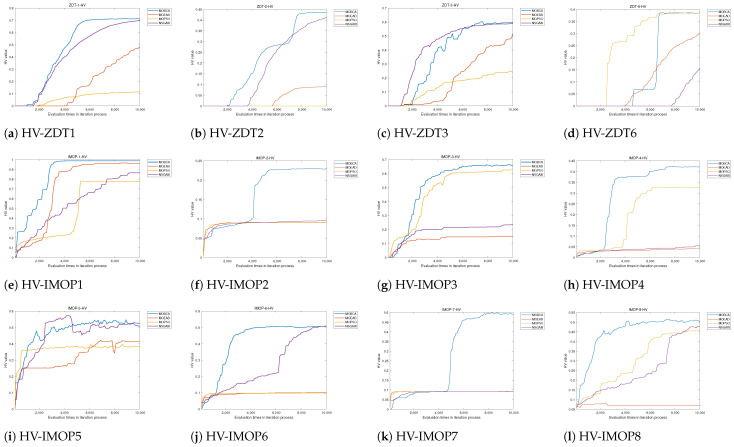
The convergence of hypervolume with the number of function evaluations.

**Figure 7:**
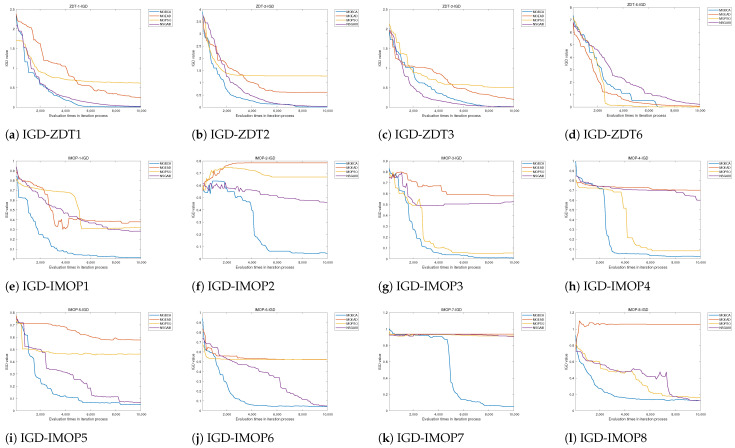
The convergence of invert generation distance with the number of function evaluations.

Utilizing the above evaluation metrics allows us to quantitatively compare MOBCA with MOPSO, NSGAIII, and MOEA/D. In addition, we can illustrate the best set of Pareto optimal solutions obtained by each algorithm on the search space. This method allows us to compare the performance of the algorithms qualitatively. All algorithms are run 30 times on the test problems, and the statistical results of these 30 runs are provided in Table 2 and Table 3. Note that we use 10,000 function evaluations for each algorithm. The qualitative results are also provided in Figure 5, Figure 6 and Figure 7.

### 4.2. Experimental Results and Discussion

The results of algorithms on the test functions are presented in Table 2 and Table 3, and the best Pareto optimal fronts obtained by all algorithms are illustrated in Figure 5. At the same time, the tracking results of HV and IGD during the iteration process are shown in Figure 6 and Figure 7.

#### 4.2.1. Results on ZDT Test Suite

Table 2 and Table 3 show that the MOBCA outperforms other algorithms in three of five ZDT test problems. The superiority can be seen in the columns, showing higher accuracy and better robustness of the MOBCA compared to others in ZDT1, ZDT3, and ZDT6.

The shape of the Pareto optimal fronts obtained by the four algorithms on ZDT1, ZDT2, ZDT3, and ZDT6 is illustrated in Figure 5a–d. Inspecting these figures, it may be observed that NSGAIII shows the poorest convergence despite its good coverage in ZDT6. However, the NSGAIII and MOBCA both provide very good convergence toward all true Pareto optimal fronts. The most interesting pattern is that the Pareto optimal solutions obtained by NSGAIII show higher coverage than MOBCA on ZDT2. However, the coverage of the MOBCA on ZDT3 is better than NSGAIII. This shows that the MOBCA has the potential to outperform NSGAIII in finding a Pareto optimal front with separate regions.

The convergence curve of HV and IGD is presented in Figure 6a–d and Figure 7a–d. Figure 6a,b and Figure 7a,b show that the MOBCA converge to the Pareto front is faster than that of NSGAIII. This shows the MOBCA can quickly converge and cover the Pareto front in comparison to NSGAIII. However, the MOBCA only demonstrates a weak advantage on ZDT3 compared to NSGAIII. We can draw a conclusion based on Table 2: although the MOBCA shows a weak advantage on ZDT3, it is a stable and robust way to solve separated MOPs.

#### 4.2.2. Results on IMOP Test Suite

The previous section investigated the performance of the proposed MOBCA on the ZDT test set. Most of the test functions in this suite are not multi-modal. To benchmark the performance of the proposed algorithm’s more challenging test set, this subsection employs IMOP benchmark functions. These functions are the most difficult test functions in the literature on multi-objective optimization and are able to confirm whether the superiority of the MOBCA is significant or not.

Inspecting the results in Table 2, it is evident that the MOBCA outperforms the MOEA/D, MOPSO, and NSGAIII in all of IMOP test functions. Since IGD is a good metric for benchmarking the convergence of an algorithm, these results indicate that the MOBCA has a better convergence on these benchmark functions. In order to observe the coverage of the algorithms, the HV metric provides quantitative result analysis in Table 3. The MOBCA failed to achieve the best results in IMOP1. We noticed that the MOBCA obtained the worst standard deviation on IMOP1 regarding HV. This means that in 30 rounds of experiments, the HV obtained by MOBCA is unstable. This may be the local optima that causes the convergence of MOBCA to stagnate.

Figure 5e–l shows the obtained Pareto front using algorithms in the IMOPs. The figures show that the MOBCA is closer to the true Pareto front, and the coverage is broader than other algorithms. Especially in Figure 5f–i,k, the MOBCA demonstrates overwhelming advantages in the coverage of the Pareto front. This means that the MOBCA can provide decision-makers with more high-quality solutions when solving practical problems. Although the MOBCA provides similar results to NSGAIII when solving IMOP5, as shown in Figure 5i, the MOBCA is significantly better than NSGAIII when solving IMOP7, as shown in Figure 5k.

The convergence speed of the MOBCA and the obtained Pareto front coverage can be reflected by the HV and IGD. Figure 6 and Figure 7 demonstrate that the MOBCA converges to the Pareto optimal front more quickly in most of the IMOPs. In particular, Figure 6f,k and Figure 7f,k present that the MOBCA can avoid the local optima effectively compared with other algorithms. The MOBCA has been developed based on the single-objective BCA, so it inherits the excellent convergence speed of the BCA. Figure 6e,h,l and Figure 7e,h,l prove this view.

#### 4.2.3. Results on Real-World Problems

The last part of Table 2 and Table 3 present real-world multi-objective optimization problems (RWMOPs). The MOBCA shows outstanding results in terms of the IGD metric, while failed archives show good results in HV. Due to the complexity and dynamics of real-life problems, the MOBCA initially designed to solve multi-objective problems may have certain flaws. However, it is still undeniable that the MOBCA can provide high-quality solutions compared with other algorithms with its current performance.

#### 4.2.4. Discussion

The qualitative and quantitative results show that the MOBCA benefits from high convergence and coverage. The high convergence of the MOBCA is inherited from the BCA. The main mechanisms that guarantee convergence in the BCA and MOBCA are the besiege and conquer mechanism. These two mechanisms emphasize exploitation and convergence proportional to the number of iterations. Since we select one solution from the archive in every iteration for each army and require the soldiers to move around the armies in the MOBCA, being trapped by local optima might be a concern. However, the results prove that the MOBCA algorithm does not suffer from local optima.

## 5. Conclusions and Future Work

This paper proposes a multi-objective version of the population optimization algorithm BCA. By introducing the grid mechanism, archive mechanism, and leader selection mechanism, and redesigning these mechanisms into the single-objective BCA algorithm, a new multi-objective algorithm, the MOBCA, is generated, which enables the BCA to deal with multi-objective optimization problems.

The proposed algorithm, the MOBCA, is compared with several excellent multi-objective optimization algorithms. These include those inspired by single-objective algorithms: MOPSO, MOEA/D, and NSGAIII. Our experimental results show that the MOBCA is superior to all compared algorithms in this paper in terms of convergence. Furthermore, the archive mechanism and grid mechanism ensure the diversity of the distribution of Pareto optimal solutions obtained by the MOBCA.

Based on the experimental results, the MOBCA shows a similar convergence speed to the BCA. It can converge to the Pareto front effectively in some MOPs. The diversity in solutions is also better than in compared algorithms except in real-world problems. This also shows that the proposed MOBCA still has certain flaws in terms of diversity, and there is considerable room for improvement.

For future work, a new multi-objective optimization mechanism should be introduced to ensure the diversity of the distribution of Pareto optimal solutions obtained by the algorithm. At the same time, a new position update formula should be considered because the MOBCA failed to determine a sufficient number of Pareto optimal solutions in some test functions.

## Figures and Tables

**Table 1:** Classical and novel multi-objective optimization algorithms.

Abbreviations	Algorithms	Authors and Year
MOGWO	Multi-Objective Grey Wolf Optimizer	Mirjalili et al., 2016 [8]
MOPSO	Multi-Objective Particle Swarm Optimization	Coello and Lechuga, 2002 [19]
MOALO	Multi-Objective Ant Lion Optimizer	Mirjalili et al., 2017 [11]
MOWOA	Multi-Objective Whale Optimization Algorithm	Mirjalili and Lewis, 2016 [20]
MOGOA	Multi-Objective Grasshopper Optimization Algorithm	Mirjalili et al., 2018 [5]
MOGA	Multi-Objective Genetic Algorithm	Konak et al., 2006 [9]
MOSOA	Multi-Objective Seagull Optimization Algorithm	Dhiman et al., 2021 [21]
NSGA	Non-Dominated Sorting Genetic Algorithm	Srinivas and Deb, 1994 [22]
NSGAII	Non-Dominated Sorting Genetic Algorithm II	Deb et al., 2002 [23]
NSGAIII	Non-Dominated Sorting Genetic Algorithm III	Deb and Jain, 2013 [24]
DN_NSGAII	Decision Space-Based Niching NSGAII	Liang et al., 2016 [25]
MOEA/D	Multi-Objective Evolutionary Algorithm based on Decomposition	Zhang and Li, 2007 [26]
RVEA	Reference Vector-Guided Evolutionary Algorithm	Cheng et al., 2016 [27]
NPGA	Niched Pareto Genetic Algorithm	Horn et al., 1994 [28]
SPEA2	Improving the Strength of the Pareto Evolutionary Algorithm	Zitzler et al., 2001 [29]
VEGA	Vector-Evaluated Genetic Algorithm	Schaffer, 2014 [30]

**Table 2:** Result of invert generation distance (IGD) after 30 experiment runs.

Problem	M	D	MOEAD	MOPSO	NSGAIII	MOBCA
IMOP1	2	10	$3.6567\times 10^{-1}$ ($5.55\times 10^{-3}$) −	$4.9790\times 10^{-1}$ ($2.62\times 10^{-1}$) −	$2.0357\times 10^{-1}$ ($7.16\times 10^{-2}$) −	$9.4338\times 10^{-2}$ ($2.43\times 10^{-1}$)
IMOP2	2	10	$7.8495\times 10^{-1}$ ($1.07\times 10^{-4}$) −	$5.9846\times 10^{-1}$ ($1.35\times 10^{-1}$) −	$4.9146\times 10^{-1}$ ($8.38\times 10^{-2}$) −	$1.8948\times 10^{-1}$ ($2.33\times 10^{-1}$)
IMOP3	2	10	$5.6532\times 10^{-1}$ ($6.24\times 10^{-2}$) −	$2.2377\times 10^{-1}$ ($2.50\times 10^{-1}$) −	$4.9451\times 10^{-1}$ ($9.06\times 10^{-2}$) −	$5.0760\times 10^{-2}$ ($1.21\times 10^{-1}$)
IMOP4	3	10	$6.1844\times 10^{-1}$ ($1.28\times 10^{-1}$) −	$5.0946\times 10^{-1}$ ($2.95\times 10^{-1}$) −	$2.8441\times 10^{-1}$ ($1.83\times 10^{-1}$) −	$2.4192\times 10^{-2}$ ($5.30\times 10^{-3}$)
IMOP5	3	10	$5.6834\times 10^{-1}$ ($5.38\times 10^{-2}$) −	$6.1207\times 10^{-1}$ ($1.58\times 10^{-1}$) −	$7.9371\times 10^{-2}$ ($3.94\times 10^{-2}$) =	$6.3583\times 10^{-2}$ ($8.71\times 10^{-3}$)
IMOP6	3	10	$3.5418\times 10^{-1}$ ($2.17\times 10^{-1}$) −	$4.9380\times 10^{-1}$ ($1.63\times 10^{-1}$) −	$1.3460\times 10^{-1}$ ($1.69\times 10^{-1}$) =	$4.7965\times 10^{-2}$ ($2.75\times 10^{-3}$)
IMOP7	3	10	$9.3885\times 10^{-1}$ ($4.21\times 10^{-5}$) −	$9.2395\times 10^{-1}$ ($1.56\times 10^{-2}$) −	$8.9101\times 10^{-1}$ ($4.29\times 10^{-2}$) −	$2.3145\times 10^{-1}$ ($3.28\times 10^{-1}$)
IMOP8	3	10	$1.0612\times 10^{0}$ ($5.61\times 10^{-3}$) −	$1.7820\times 10^{-1}$ ($2.63\times 10^{-2}$) −	$1.6625\times 10^{-1}$ ($1.53\times 10^{-1}$) −	$1.2358\times 10^{-1}$ ($7.28\times 10^{-3}$)
ZDT1	2	30	$1.3888\times 10^{-1}$ ($6.42\times 10^{-2}$) −	$5.5105\times 10^{-1}$ ($1.02\times 10^{-1}$) −	$1.7452\times 10^{-2}$ ($3.26\times 10^{-3}$) −	$9.7979\times 10^{-3}$ ($1.06\times 10^{-3}$)
ZDT2	2	30	$5.2995\times 10^{-1}$ ($6.18\times 10^{-2}$) −	$1.3942\times 10^{0}$ ($2.39\times 10^{-1}$) −	$3.4393\times 10^{-2}$ ($3.23\times 10^{-2}$) +	$9.9316\times 10^{-2}$ ($2.36\times 10^{-1}$)
ZDT3	2	30	$1.5911\times 10^{-1}$ ($4.00\times 10^{-2}$) −	$4.4792\times 10^{-1}$ ($7.11\times 10^{-2}$) −	$1.8192\times 10^{-2}$ ($8.71\times 10^{-3}$) −	$1.2143\times 10^{-2}$ ($1.45\times 10^{-3}$)
ZDT4	2	10	$5.4634\times 10^{-1}$ ($2.04\times 10^{-1}$) +	$1.0012\times 10^{1}$ ($4.40\times 10^{0}$) +	$5.5155\times 10^{-1}$ ($3.00\times 10^{-1}$) +	$2.4867\times 10^{1}$ ($1.61\times 10^{1}$)
ZDT6	2	10	$7.4800\times 10^{-2}$ ($2.16\times 10^{-2}$) −	$2.4192\times 10^{-1}$ ($3.75\times 10^{-1}$) =	$1.6163\times 10^{-1}$ ($6.23\times 10^{-2}$) −	$6.9473\times 10^{-3}$ ($8.46\times 10^{-4}$)
RWMOP5	2	4	NaN (NaN) *	NaN (NaN)	$1.8897\times 10^{0}$ ($4.60\times 10^{-3}$) =	$1.8888\times 10^{0}$ ($3.33\times 10^{-4}$)
RWMOP9	2	4	$1.6484\times 10^{3}$ ($5.43\times 10^{-2}$) −	$9.8115\times 10^{8}$ ($4.39\times 10^{9}$) −	$1.3867\times 10^{1}$ ($4.16\times 10^{1}$) −	$2.7131\times 10^{0}$ ($7.46\times 10^{0}$)
RWMOP11	5	3	$3.6986\times 10^{6}$ ($3.50\times 10^{4}$) −	$4.2657\times 10^{6}$ ($1.21\times 10^{6}$) −	$2.5476\times 10^{6}$ ($1.84\times 10^{4}$) −	$2.4764\times 10^{6}$ ($8.51\times 10^{4}$)
RWMOP14	2	5	NaN (NaN)	$1.8521\times 10^{6}$ ($5.86\times 10^{6}$) −	$1.1596\times 10^{-1}$ ($1.48\times 10^{-1}$) −	$1.2783\times 10^{-2}$ ($3.44\times 10^{-3}$)
RWMOP16	2	2	NaN (NaN)	$1.1851\times 10^{8}$ ($4.93\times 10^{8}$) −	$1.9990\times 10^{-3}$ ($3.21\times 10^{-7}$) =	$1.9989\times 10^{-3}$ ($1.32\times 10^{-18}$)
+/−/=			1/14/0	1/15/1	2/12/4	

* NaN indicates that no feasible solution was found. “+” and “−” indicate the number of test problems in which the compared algorithm shows significantly better performance of worse performance than MOBCA respectively. The symbol “=” indicates there is no significant difference between MOBCA and compared algorithms.

**Table 3:** Result of hypervolume (HV) after 30 experimental runs.

Problem	M	D	MOEAD	MOPSO	NSGAIII	MOBCA
IMOP1	2	10	$9.6578\times 10^{-1}$ ($1.71\times 10^{-3}$) +	$4.6753\times 10^{-1}$ ($3.29\times 10^{-1}$) −	$9.4891\times 10^{-1}$ ($4.17\times 10^{-2}$) +	$8.9643\times 10^{-1}$ ($2.74\times 10^{-1}$)
IMOP2	2	10	$9.0908\times 10^{-2}$ ($3.87\times 10^{-7}$) −	$9.3116\times 10^{-2}$ ($2.54\times 10^{-2}$) −	$9.5469\times 10^{-2}$ ($8.72\times 10^{-3}$) −	$1.8745\times 10^{-1}$ ($6.33\times 10^{-2}$)
IMOP3	2	10	$1.7102\times 10^{-1}$ ($5.18\times 10^{-2}$) −	$4.8640\times 10^{-1}$ ($2.05\times 10^{-1}$) −	$2.7823\times 10^{-1}$ ($5.04\times 10^{-2}$) −	$6.2497\times 10^{-1}$ ($1.05\times 10^{-1}$)
IMOP4	3	10	$6.5316\times 10^{-2}$ ($4.14\times 10^{-2}$) −	$1.2923\times 10^{-1}$ ($1.37\times 10^{-1}$) −	$2.1154\times 10^{-1}$ ($1.02\times 10^{-1}$) −	$4.2134\times 10^{-1}$ ($6.92\times 10^{-3}$)
IMOP5	3	10	$4.2214\times 10^{-1}$ ($2.69\times 10^{-2}$) −	$3.2787\times 10^{-1}$ ($9.24\times 10^{-2}$) −	$5.2323\times 10^{-1}$ ($1.77\times 10^{-2}$) =	$5.2656\times 10^{-1}$ ($1.23\times 10^{-2}$)
IMOP6	3	10	$2.4057\times 10^{-1}$ ($1.82\times 10^{-1}$) −	$1.9050\times 10^{-1}$ ($1.28\times 10^{-1}$) −	$4.4783\times 10^{-1}$ ($1.21\times 10^{-1}$) =	$5.0203\times 10^{-1}$ ($3.50\times 10^{-3}$)
IMOP7	3	10	$9.0909\times 10^{-2}$ ($2.22\times 10^{-7}$) −	$9.1362\times 10^{-2}$ ($7.74\times 10^{-4}$) −	$9.4667\times 10^{-2}$ ($5.23\times 10^{-3}$) −	$4.0964\times 10^{-1}$ ($1.54\times 10^{-1}$)
IMOP8	3	10	$7.0160\times 10^{-2}$ ($1.92\times 10^{-3}$) −	$4.5644\times 10^{-1}$ ($2.55\times 10^{-2}$) −	$4.7606\times 10^{-1}$ ($4.79\times 10^{-2}$) −	$5.0825\times 10^{-1}$ ($1.63\times 10^{-2}$)
ZDT1	2	30	$5.7239\times 10^{-1}$ ($5.19\times 10^{-2}$) −	$1.6321\times 10^{-1}$ ($6.64\times 10^{-2}$) −	$6.9910\times 10^{-1}$ ($4.47\times 10^{-3}$) −	$7.1044\times 10^{-1}$ ($1.71\times 10^{-3}$)
ZDT2	2	30	$1.0100\times 10^{-1}$ ($1.58\times 10^{-2}$) −	$0.0000\times 10^{0}$ ($0.00\times 10^{0}$) −	$4.0131\times 10^{-1}$ ($3.12\times 10^{-2}$) +	$3.8379\times 10^{-1}$ ($1.36\times 10^{-1}$)
ZDT3	2	30	$5.9559\times 10^{-1}$ ($6.32\times 10^{-2}$) =	$2.9460\times 10^{-1}$ ($5.23\times 10^{-2}$) −	$5.9328\times 10^{-1}$ ($2.29\times 10^{-2}$) −	$5.9629\times 10^{-1}$ ($3.20\times 10^{-3}$)
ZDT4	2	10	$1.6822\times 10^{-1}$ ($1.31\times 10^{-1}$) +	$0.0000\times 10^{0}$ ($0.00\times 10^{0}$) =	$2.4230\times 10^{-1}$ ($1.73\times 10^{-1}$) +	$0.0000\times 10^{0}$ ($0.00\times 10^{0}$)
ZDT6	2	10	$2.8712\times 10^{-1}$ ($2.65\times 10^{-2}$) −	$2.6527\times 10^{-1}$ ($1.57\times 10^{-1}$) −	$2.0641\times 10^{-1}$ ($5.67\times 10^{-2}$) −	$3.8490\times 10^{-1}$ ($8.60\times 10^{-4}$)
RWMOP5	2	4	NaN (NaN) *	NaN (NaN)	$4.3321\times 10^{-1}$ ($1.05\times 10^{-3}$) +	$2.6988\times 10^{-1}$ ($4.08\times 10^{-3}$)
RWMOP9	2	4	$5.3041\times 10^{-2}$ ($5.97\times 10^{-5}$) −	$6.0482\times 10^{-1}$ ($1.71\times 10^{-1}$) +	$4.0899\times 10^{-1}$ ($9.35\times 10^{-4}$) +	$4.0771\times 10^{-1}$ ($5.24\times 10^{-4}$)
RWMOP11	5	3	$5.8183\times 10^{-2}$ ($2.63\times 10^{-3}$) −	$1.9772\times 10^{-3}$ ($2.72\times 10^{-3}$) −	$9.3776\times 10^{-2}$ ($7.35\times 10^{-4}$) +	$9.2078\times 10^{-2}$ ($2.18\times 10^{-3}$)
RWMOP14	2	5	NaN (NaN)	$9.9135\times 10^{-1}$ ($5.79\times 10^{-3}$) +	$6.1549\times 10^{-1}$ ($1.49\times 10^{-3}$) +	$3.4281\times 10^{-1}$ ($7.80\times 10^{-3}$)
RWMOP16	2	2	NaN (NaN)	$1.7356\times 10^{-1}$ ($9.84\times 10^{-6}$) −	$7.6320\times 10^{-1}$ ($3.28\times 10^{-5}$) +	$7.5211\times 10^{-1}$ ($2.89\times 10^{-3}$)
+/−/=			2/12/1	2/14/1	8/8/2	

* NaN indicates that no feasible solution was found. “+” and “−” indicate the number of test problems in which the compared algorithm shows significantly better performance of worse performance than MOBCA respectively. The symbol “=” indicates there is no significant difference between MOBCA and compared algorithms.

## Data Availability

Data is contained within the article.
